# Revisiting Tumors and the Cardiovascular System: Mechanistic Intersections and Divergences in Ferroptosis

**DOI:** 10.1155/2020/9738143

**Published:** 2020-08-17

**Authors:** Yani Wang, Xiang Peng, Maomao Zhang, Ying Jia, Bo Yu, Jinwei Tian

**Affiliations:** ^1^Department of Cardiology, The Second Affiliated Hospital of Harbin Medical University, Harbin 150086, China; ^2^The Key Laboratory of Myocardial Ischemia, Harbin Medical University, Ministry of Education, Harbin 150086, China; ^3^Guangxi Key Laboratory of Diabetic Systems Medicine, Guilin Medical University, Guilin, 541000 Guangxi, China

## Abstract

Ferroptosis was recently identified as an iron-dependent regulatory necrosis process mediated by polyunsaturated fatty acid (PUFA) peroxidation. The pivotal events related to oxidative stress in ferroptosis include direct or indirect glutathione peroxidase 4 (GPX4) inhibition, ferrous iron overload, and lipid peroxidation. The links between ferroptosis and multiple pathological processes including tumor and cardiovascular system disease have become increasingly apparent, and the mechanisms and compounds involved in ferroptosis, such as reduction of coenzyme Q_10_ (ubiquinone/CoQ_10_), are gradually emerging. Current reports have revealed crossroads between ferroptosis and other multiple responses. This overview of the current research illuminates the mechanisms involving ferroptosis-related compounds and emphasizes the crosstalk between ferroptosis and other responses, including mitochondrial damage, endoplasmic reticulum stress (ER stress), autophagy, and the release of damage-associated molecular patterns (DAMPs), to reveal the intersections of regulatory mechanisms. This review also outlines the discovery, characterization, and pathological relevance of ferroptosis and notes controversial elements in ferroptosis-related mechanisms, such as nuclear factor E2-related factor 2 (Nrf2), sequestosome 1 (p62/SQSTM1), and heat shock protein family A member 5 (HSPA5). We hope our inferences will supply a partial reference for disorder prevention and treatment.

## 1. Introduction

Cell death is split into two mutually independent facets by the Nomenclature Committee on Cell Death (NCCD) in terms of a functional distinction: accidental cell death (ACD) and regulated cell death (RCD) [1]. In extremely harsh physicochemical or mechanical settings, which commonly include unadaptable pressure states, pH conditions, or shearing stimuli, cells undergo sudden, uncontrolled demise called ACD. RCD, in contrast, involves tremendous genetically encoded networks of molecular mechanisms and can therefore be regulated by genetic or pharmacologic interference. Under complete physiological regulation, RCD is also termed programmed cell death (PCD) and includes apoptosis, necrosis, and autophagy, among other pathways [1]. Ferroptosis, a form of RCD that is the focus of increasing research attention, is a type of regulatory necrosis mediated by iron-dependent and polyunsaturated fatty acid (PUFA) peroxidation, which distinguishes it from other types of PCD in terms of morphology, biochemistry, and genetics [2].

## 2. Basics of Ferroptosis

### 2.1. Discovery

In 2003, tumorigenic cells and their precursors were engineered by introducing vectors of genetic elements (e.g., ST, small T oncoproteins, and RAS^V12^, an oncogenic allele of HRAS) into primary BJ fibroblasts, and erastin was identified among 23550 compounds using synthetic lethal chemical screening and found to serve as a novel lethal inducer of selective, nonapoptotic demise in RAS^V12^- and ST-expressing cells [3]. In 2007, Yagoda et al. confirmed that antioxidants (e.g., *α*-tocopherol) prevented erastin-induced death in HRAS and KRAS cells, and a similar protective effect could be achieved by KRAS and BRAF knockdown, voltage-dependent anion channel (VDAC2/3) knockdown, or the inhibition of mitogen-activated protein kinase (MAPK)/extracellular signal-regulated kinase (ERK) kinase 1/2 (MEK1/2), demonstrating that the selective lethality of erastin caused RAS mutant tumor cell death in a RAS-RAF-MEK-dependent, oxidative, nonapoptotic manner [4]. Additionally, erastin damaged mitochondrial function by binding VDAC2/3 directly [4]. In 2008, the RAS-selective lethal compounds RSL-3 and RSL-5 were found by synthetic lethal screening with small molecules to exhibit selective lethal properties in oncogenic RAS mutant BJ cells, eliciting iron- and MEK-dependent oxidative erastin-like cell death [5]. In 2012, erastin and RSL-3 were reported to induce oxidative responses in NRAS mutant HT-1080 fibrosarcoma cells to generate lipid reactive oxygen species (ROS), and iron-dependent cell death was confirmed by coculture on erastin and various metals (e.g., Mn, Cu, and different forms of iron) [2]. Hence, Dixon et al. explicitly proposed that ferroptosis is an iron-dependent regulatory necrosis process mediated by PUFA peroxidation that can be disrupted by the specific inhibitor ferrostatin-1 (Fer-1) and distinguished from PCD by morphological, biochemical, and genetic features [2].

### 2.2. Characterization

Ferroptosis has certain characteristic features and phenotypes that distinguish it from other forms of PCD. (1) Morphologically, mitochondria after ferroptosis are shrunken in size with reduced or absent cristae and increased membrane density and accompanied by broken exterior membranes. These characteristics can be distinguished from the concentrated chromosomes occurring in apoptosis, the swollen cytoplasm and organelles and broken plasmalemma occurring in necrosis, and the double-membrane vesicles occurring in autophagy [1, 2, 6]. (2) Biochemically, the specific aspects of ferroptosis include the accumulation of ferrous irons and ROS and the reduction of cysteine and glutathione (GSH). Some characteristics that distinguish ferroptosis from apoptosis, necrosis, and autophagy, respectively, include active caspases, vast ATP wastage, and the transformation of microtubule-associated protein 1 light chain 3 (MAP1LC3) from LC3I to LC3II [1, 2, 6]. (3) Genetically, six genes specific for ferroptosis in NRAS mutant HT-1080 cells and HRAS mutant Calu-1 cells were detected using shRNA screening among 1080 mitochondrial functional genes, including RPL8, IREB2, ATP5G3, CS, TTC35, and ACSF2 [2]. Moreover, the specific inhibitors for ferroptosis (Fer-1, deferoxamine) do not affect other forms of cell death, while it is controversial whether the inhibitors of other types of cell death have the ability to regulate ferroptosis.

## 3. Oxidative Stress in Ferroptosis

On the basis of the essence and mechanism of cell death, NDCC [7] defined the term “ferroptosis” precisely in 2018 as one kind of RCD caused by GPX4 disorder in regulating oxidative imbalance in the intracellular environment that could be suppressed by iron chelators and antioxidants. This section briefly describes the multiple signaling pathways that have been shown in recent years to contribute to regulating ferroptotic progress via oxidative stress, which occurs due to imbalance between ROS generation and scavenging [8].

### 3.1. Interference in System x_C_^−^

Because glutamate- (Glu-) induced excitotoxic neuronal death can be rescued by Fer-1, the cystine/glutamate antiporter (system x_C_^−^), as a dominant target of Glu toxicity, is used to explore the mechanisms of erastin-induced ferroptosis. Sulfasalazine (SAS), an inhibitor of system x_C_^−^, had the same effect as erastin in organotypic hippocampal slice cultures (OHSCs); *β*-mercaptoethanol (*β*-ME) recovered the uptake of cystine by a nonsystem x_C_^−^ mechanism to compensate for the suppression of system x_C_^−^ by erastin, followed by degradation of cystine to cysteine to rescue ferroptosis [2, 9, 10]. System x_C_^−^ consists of SLC3A2 and SLC7A11, but the latter forms the main structure [2]. The above results verify that erastin promotes ferroptosis by inhibiting the function of system x_C_.

**Figure 1:**
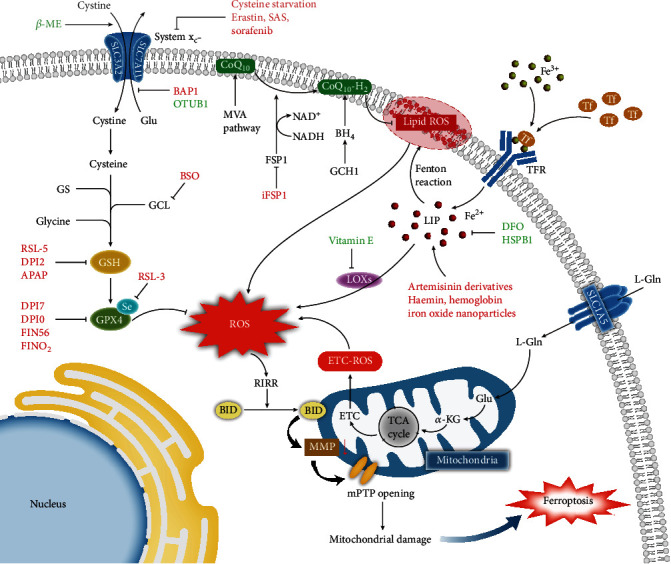
Increasing ROS triggers mitochondrial damage related to ferroptosis under cysteine starvation. The pathways correlated with reactive oxygen species (ROS) accumulation are as follows. (1) Cystine turns into cysteine after entering the cytoplasm by system x_C_^−^; the latter involves glutathione (GSH) biosynthesis with the catalysis of *γ*-glutamylcysteine ligase (GCL), glutathione synthetase (GS), and glycine; and GSH as a cofactor further facilitates the scavenging of ROS by glutathione peroxidase 4 (GPX4). (2) Ferric irons transferred by transferrin (Tf) become ferrous irons after entering the cytoplasm by the transferrin receptor (TFR); the ferrous irons enrich the labile iron pool (LIP), which facilitates the generation of ROS via the Fenton reaction and enzymatic reaction (e.g., LOXs). (3) L-Glutamine (L-Gln) becomes ketoglutaric acid (*α*-KG) via glutaminolysis after entering the cytoplasm by solute carrier family 1 member 5 (SLC1A5), and *α*-KG supplies a carbon source for the tricarboxylic acid (TCA) cycle, which activates the electron transfer chain (ETC) to generate ETC-ROS. (4) The coenzyme Q_10_ (ubiquinone/CoQ_10_) derived from the mevalonate pathway (MVA pathway) and its reduced form ubiquinol (CoQ_10_-H_2_) generated via the NADH-FSP1-CoQ_10_ pathway and the GCH1-BH_4_-phospholipid axis participate in the scavenging on lipid ROS. The increase in ROS triggers ROS-induced ROS release (RIRR), which promotes proapoptotic protein BID translocation into the mitochondria. Then, the collapse of mitochondrial membrane potential (MMP) and the sustained opening of the mitochondrial permeability transition pore (mPTP) cause mitochondrial damage, inducing ferroptosis. Inducers of ferroptosis are in red; inhibitors of ferroptosis are in green. Relevant abbreviations: NADH: nicotinamide adenine dinucleotide; FSP1: ferroptosis suppressor protein 1; GCH1: GTP cyclohydrolase-1; BH_4_: tetrahydrobiopterin.

Furthermore, metabolomic profiling detected that GSH levels were diminished downstream of erastin-induced ferroptosis, leading to glutathione peroxidase 4 (GPX4) inactivation because GPX4 required GSH as a crucial cofactor [11–13]. Buthionine sulfoximine (BSO), the *γ*-glutamylcysteine ligase (GCL) inhibitor that obstructs GSH biosynthesis, worsens GSH levels and causes aggravated lipid ROS accumulation, promoting ferroptosis [12, 14]. In addition, glutathione synthetase (GS) and glycine were also indispensable to the process by which cysteine is metabolized to GSH [12, 14, 15]. In summary, the decrease in cysteine due to system x_C_^−^ suppression affects GSH biosynthesis; subsequently, lipid peroxidation occurs because GPX4 cannot reduce ROS accumulation poorly, which underlies the initiation of ferroptosis by erastin-induced oxidative stress (Figure 1).

Numerous compounds act as ferroptosis regulators, including exogenic small molecules (e.g., erastin), clinical drugs (e.g., sorafenib), nanosized materials (e.g., iron oxide nanoparticles, FePt nanoparticles), and organics (e.g., vitamin E). In the modulatory profiling strategy, the modulatory effect (Me) values showed that sorafenib could enhance the lethality of erastin, and *β*-ME and Fer-1 could reverse the effects of sorafenib, such as lipid ROS accumulation and total GSH decline [16]. This means that sorafenib initiates ferroptosis by limiting cysteine importation, similar to erastin and SAS. In contrast, cysteinyl-tRNA synthetase (CARS) knockdown prompted the accumulation of cystathionine via activation of the transsulfuration pathway so that ferroptosis was discontinued [17]. BRCA1-associated protein 1 (BAP1) [18], the crucial constituent of the deubiquitinase complex, attenuated the initiation and extension of SLC7A11 transcription by deubiquitinating histone 2A, while the steady-state levels and half-life of SLC7A11 were improved by combination with the ovarian tumor family member deubiquitinase (OTUB1) [19]. Ultimately, ferroptosis occurred in response to downregulate the stability or the expression of SLC7A11. It may well be speculated that BAP1 plays a part in the basal expression of SLC7A11, while OTUB1 participates in maintenance after basal expression. Interference with the function of system x_C_^−^ or cysteine metabolism by the above compounds initiates ferroptosis, suggesting that the vital role of system x_C_^−^ consists of interdicting ferroptosis.

### 3.2. Direct or Indirect GPX4 Inhibition

RSL-induced ferroptosis is classified into 2 types. Metabolomic profiling shows that in type I, RSL-5, which belongs to type I, targets VDAC2/3 and system x_C_^−^ to reduce GSH biosynthesis, indirectly impairing GPX4, similar to erastin. Chemoproteomics shows that RSL-3, in type II, combines with selenocysteine (Sec) in the active site of GPX4, directly resisting antioxidation [5, 12]. Both direct and indirect mechanisms of weakening GPX4 promote the accumulation of ROS by disrupting the function of GPX4 which converts toxic peroxidative production into nontoxic lipid alcohol, ultimately resulting in oxidative imbalance and ferroptosis [12].

Observation of the rescue effects of Fer-1 against various doses of acetaminophen (APAP) in primary hepatocytes demonstrated a remarkable protective effect at a higher APAP concentration [20]. On the other hand, it did not effectively promote APAP-induced ferroptosis in HepG2 cells because the HepG2 cells maintained GSH levels, failing to generate the APAP metabolite N-acetyl-p-benzoquinone imine (NAPQI) due to the lack of phase 1 enzymes [20]. Briefly, APAP overdose induces ferroptosis by depleting GSH levels. Analyzing GPX4 activity by adding the exogenous GPX4 substrate phosphatidylcholine hydroperoxide (PC-OOH) showed that DPI7 and DPI10 limited GPX4 activity but not GSH expression, unlike DPI2 [12]. According to reports, an essential step in the formation of the Sec-containing activated site for GPX4 is the generation of mature selenocysteine tRNA (Sec-tRNA) by tRNA isopentenyl transferase via the mevalonate pathway (MVA pathway) and subsequent integration of Sec into the selenoprotein GPX4 [21, 22]. FIN56 decreased the abundance of GPX4 at the posttranslational level rather than by GSH depletion; moreover, the accumulation of ROS in cells treated with FIN56 seemed slower than in cells treated with RSL-3, and the former was obstructed by fatty acid metabolism-related acetyl-CoA carboxylase (ACC) inhibitors [23]. That is, FIN56 requires ACC activity to degenerate GPX4 protein instead of ferroptosis-induced pathways such as RSL-3 or the depletion of GSH. In addition, FIN56 enhances the activity of squalene synthase (SQS), which upregulates cholesterol synthesis via the MVA pathway to induce ferroptosis [24]. The mechanism by which endoperoxide-containing 1,2-dioxolane (FINO_2_) attenuates GPX4 activity differs from the mechanisms of the direct GPX4 inhibitor RSL-3 and the inhibitor FIN56 for GPX4 protein abundance or the GSH depletion [24].

Overall, APAP, DPI2, and RSL-5 negatively regulate GSH biosynthesis to constrict GPX4 indirectly; conversely, DPI7, DPI10, RSL-3, and immature Sec-tRNA inactivate GPX4 directly. FINO_2_ promotes ferroptosis by attenuating GPX4 activity, similar to RSL-3 and FIN56, but the mechanism is distinct. The active site, cofactor, and posttranslational regulation of GPX4 greatly influence the balance between ROS and antioxidant defenses to prevent ferroptosis. To date, how FINO_2_ inactivates GPX4 has not been revealed.

### 3.3. Iron Metabolism

Transferrin (Tf) transports ferric irons into various tissues and cells by combining with transferrin receptor (TFR) to act on multifarious reactions after releasing free iron, while the surplus intracellular iron is exported by ferroportin or stored in ferritin in the form of ferric irons [25]. The above summarizes the physiological process of iron metabolism. Pathologically, excess iron increases the quantity of ferrous irons; the increase in the labile iron pool (LIP) subsequently induces oxidative stress via the Fenton reaction and the activity of ROS-generating enzymes ROS (e.g., lipoxygenases), leading to ferroptosis [26, 27] (Figure 1).

In light of reports concerning mechanisms of iron overload-induced ferroptosis, under ferric citrate or high-iron diet treatment, Tf tended to be saturated and failed to bind excess iron, causing an increase in ferrous irons that propelled the oxidation of cysteine to cystine. Subsequently, ROS accumulated due to the attenuation of GSH synthesis, which decreased the cytosolic thiol that stabilizes KEAP1/Nrf2 heterodimers. After the heterodimers dissociated, nuclear factor E2-related factor 2 (Nrf2) transited from the cytoplasm into the nucleus, positively regulating the expression of the ferroptotic protective gene SLC7A11 [28]. Intriguingly, doxorubicin (DOX) induced Nrf2 to accumulate in the nucleus and then initiated the transcription of antioxidant response elements (AREs), including HO-1; thereafter, HO-1 decomposed, causing iron overload, which in turn caused lipid peroxidation and iron-replete ferroptosis [29]. The paradoxical effects of Nrf2 have yet to be elucidated. As Nrf2 also modulates genes related to lipid homeostasis, the Nrf2-lipid peroxidation-ferroptosis axis is currently being gradually but incompletely established in diseases [30]. Hence, Nrf2 lies at the intersection of iron homeostasis, lipid homeostasis, and redox homeostasis, which urges us to seek the precise niche of Nrf2 in ferroptosis and then explore the potential for improvement in disorders relating to ferroptosis.

Artemisinin [31] derivatives, which rely on ferrous iron to determine their activity, regulate the expression of iron metabolism genes such as TFR, the RNA-binding protein IREB2, and specific ferroptosis-related genes. Liver ferroptosis can be rescued by SLC39A14 knockout in hepatocyte-specific Tf knockout mice [32]. Heat shock protein family B member 1 (HSPB1) [33] protects the actin cytoskeleton to obstruct iron uptake. CDGSH iron sulfur domain 1 (CISD1) [34] is an iron export protein of mitochondria that can prevent ferroptosis. In short, the endogenous inhibition of HSPB1 and CISD1 or changes in the expression of iron metabolism genes can trigger oxidative stress and iron-replete ferroptosis. Likewise, exogenous iron ions obtained from haemin [35], hemoglobin [36], and iron-containing nanoparticles [37] also facilitate iron overload-induced ferroptosis.

### 3.4. Lipid Peroxidation

Lipid peroxidation as a result of oxidative stress plays a pivotal role in executing ferroptosis. PUFAs, the primary substrate for iron-catalyzed lipid peroxidation, insert phospholipids under the action of lysophosphatidylcholine acyltransferase 3 (LPCAT3) after the acetylation of acyl-CoA synthetase long-chain family member 4 (ACSL4) [38]. Toxic ROS generated by lipoxygenases (LOXs) or the Fenton reaction induce the loss of membranal stability and integrity, the formation of lipid pores and micelles in the cytomembrane, and the release of noxious aldehydes (e.g., 4-hydroxy-2-nonenals (4-HNEs) and malondialdehydes (MDAs)) [14]. In summary, the essential elements of lipid peroxidation are ROS accumulation and substrate PUFA rising.

In the pentose phosphate pathway (PPP), glucose is metabolized to nicotinamide adenine dinucleotide phosphate (NADPH), which is then oxidized into ROS by NADPH oxidase (NOX) [2]. Ketoglutaric acid (*α*-KG) from glutaminolysis interacts with the tricarboxylic acid (TCA) cycle, oxidative phosphorylation, and citrate-associated fatty metabolism to augment ROS and lipid synthesis [39–41]. Additionally, acetyl-CoA is metabolized into cholesterol via HMG-CoA reductase in the MVA pathway [23]. In summary, the two facets causing ROS accumulation are a decrease in elimination and an increase in generation. Weakened antioxidant defenses cannot sufficiently eliminate ROS, and on the other hand, the NOX/PPP pathway, LOXs, and *α*-KG promote ROS generation. In addition, cholesterol biosynthesis impelled by the MVA pathway and fatty metabolism propelled by *α*-KG provide excess oxidative substrates for producing ROS.

In response to erastin treatment or extracellular matrix detachment, the *α*6*β*4 integrin-Src signaling axis was activated to phosphorylate signal transducer and activator of transcription 3 (STAT3), which diminished ACSL4 expression [42]. According to the existing literature, the clinical antidiabetic medicine thiazolidinediones (TZNs) showed a rescue effect on ferroptosis by targeting and inhibiting ACSL4 [43]. ACSF and CS, regulators of fatty acid metabolism in mitochondria, have been found to participate in ferroptosis by providing lipid precursors [2]. Additionally, the introduction of deuterium PUFAs (D-PUFAs) instead of natural PUFAs retarded or vanished the generation of lipid peroxides induced by erastin and RSL-3 [27]. Alternatively, the occurrence of iron-dependent lipid peroxidation could be rescued by chelation with deferoxamine (DFO) [28]. The antioxidant vitamin E family, consisting of tocotrienols and tocopherols, repressed LOX activity to rescue ferroptosis [44]. Similarly, the protectors Fer-1 and liproxstatin-1 can rescue ferroptosis through their antioxidant effects [45].

In summary, DFO mitigates LIP, which correlates with spontaneous or enzymatic lipid peroxidation. TZNs, *α*6*β*4 integrin, D-PUFAs, and fatty acid metabolism-associated genes ACSF2 and CS limit the substrates for peroxidation. Antioxidants (e.g., vitamin E family, Fer-1, and liproxstatin-1) retard the storage of ROS. All of these factors inhibit ferroptosis by regulating lipid peroxidation after oxidative stress.

### 3.5. Reduction of CoQ_10_

Attention is currently focused on ferroptosis resistance in oncotherapy, particularly the role of coenzyme Q_10_ (ubiquinone/CoQ_10_), an electron carrier functioning in the mitochondrial respiratory chain and as an antioxidant for scavenging free radicals [46].

CoQ_10_ synthesized via the MVA pathway is converted into the reduced form ubiquinol (CoQ_10_-H_2_) by the oxidoreductase ferroptosis suppressor protein 1 (FSP1), which uses NADH as a cofactor, to suppress lipid peroxidation and induce ferroptosis resistance [47–50]. In this process, myristoylation of a specific N-terminal sequence of FSP1 is essential for the localization of FSP1 in the plasma membrane to exert its catalytic effect [49]. Notably, H460 lung cancer cells with high expression of FSP1 survived normally upon GPX4 knockout, whereas rapid death was observed in GPX4 and FSP1 double-knockout cells. In addition, no obvious variation of ACSL4 and GSH expression was observed after overexpressing FSP1 [48]. That is, the NADH-FSP1-CoQ_10_ pathway is independent of the classic ferroptosis-related mechanism but acts synergistically with GPX4 to inhibit ferroptosis (Figure 1).

The GCH1-BH_4_-phospholipid axis also acts in ferroptosis resistance via reduction of CoQ_10_. GTP cyclohydrolase-1 (GCH1) overexpression in MF cells selectively and obviously enhances the biosynthesis of tetrahydrobiopterin (BH_4_) to enrich CoQ_10_-H_2_ and then protects two-tailed PUFA phosphatidylcholines from peroxidation [50, 51]. Similar to the NADH-FSP1-CoQ_10_ pathway, this axis does not rely on known ferroptosis-related proteins. Strikingly, the protective effects of GCH1 for specific lipids demonstrate that two-tailed PUFA phosphatidylcholines play critical roles in the response to peroxidation and executing ferroptosis [51] (Figure 1).

In addition to CoQ_10_-H_2_, the endosomal sorting complexes required for transport- (ESCRT-) III-mediated membrane repair mechanism defends against lipid peroxidation and ferroptosis through membrane budding and fission. ESCRT-III depends on FSP1 for recruitment to the plasma membrane via the FSP1-dependent ESCRT-III pathway [52].

These pathways have provided new insights on ferroptosis resistance under oxidative stress. For lipid peroxidation, three factors may suppress the process: (1) the decrease in oxidative substrates, including the inhibition of lipid synthesis and insertion; (2) the enhancement of antioxidative defense associated with the classic GPX4 and novel CoQ_10_-H_2_ pathways; and (3) the repair of membrane integrity after oxidative damage. For oncotherapy, these pathways offer compensatory or parallel effects for ferroptosis resistance mediated by GPX4, and the molecules involved hold potential as biomarkers for evaluating drug resistance and selecting chemotherapeutics. The discovery of a potent FSP1 inhibitor iFSP1 [48] is important to exploit the methods for treatment and research. Furthermore, the protection of adjacent cells mediated by membrane-permeable BH_4_ and the dense three-dimensional spheroids induced by GCH1 further supplement ferroptosis resistance in tumor progression [51].

## 4. Crosstalk Related to Ferroptosis

### 4.1. ETC-ROS Induction and Mitochondrial Damage

Dixon et al. used rotenone to induce the accumulation of electron transfer chain-associated ROS (ETC-ROS), causing mitochondrial dysfunction in HT-1080 cells. However, ETC-ROS levels did not decrease after administration of DFO. Moreover, erastin-induced ferroptosis was not blocked in designed 143B*ρ*^0^ cells that lacked genes related to mitochondrial function. The ultimate conclusion was that erastin-induced lipid ROS accumulation differed from ETC-ROS accumulation [2]. Nevertheless, recent studies have produced different results, finding that ferroptosis also accompanies ETC-ROS accumulation and mitochondrial damage.

Gao et al. used fractionation, purification, and mass spectrometry analysis to explore why fetal bovine serum (FBS) promotes ferroptosis under cysteine starvation. The mitochondrial oxidative phosphorylation inhibitor oligomycin was found to rescue serum-dependent ferroptosis, and the requirement for Tf and L-glutamine (L-Gln) in serum-dependent ferroptosis was verified [39]. Consistent with these findings, mitochondrial depletion reversed the ferroptosis induced by cysteine starvation [41]. Mechanistically, under cysteine starvation, the degradation of Gln, which enters cells via solute carrier family 1 member 5 (SLC1A5), into *α*-KG via glutaminolysis supplies an abundant carbon source for the TCA cycle, which ultimately activates ETC to induce ETC-ROS accumulation and mitochondrial membrane potential (MMP) abnormality [41]. The change in MMP from hyperpolarization to collapse causes the mitochondrial permeability transition pore (mPTP) to remain persistently open and thereby damages the mitochondria [41] (Figure 1).

As mentioned above, ROS, which originate from iron overload, GSH depletion, and ETC-ROS, trigger ROS-induced ROS release (RIRR) and consequently very high ROS accumulation, a phenomenon that is also observed in iron-treated endothelial cells [41, 53, 54]. On account of the reverse effect of the proapoptosis protein BID inhibitor BI6c9 in increasing ROS and mitochondrial fragmentation as well as decreasing MMP and ATP levels in erastin-treated neuronal HT-22 cells, Neitemeier et al. substantiated that excess ROS promoted BID transactivation to let BID translocate into mitochondria, ultimately leading to the collapse of MMP, the sustained opening of mPTPs, and mitochondrial damage [55]. Mitochondrial dysfunction was also deemed to be a related factor in whole cigarette smoke condensate- (WCSC-) induced ferroptosis [56]. In addition, the promotion of the progression of DOX-induced cardiotoxicity by mitochondria-dependent ferroptosis was verified [57]. In brief, iron overload, GSH depletion, and ETC-ROS induced by cysteine starvation drive an ROS burst that causes mitochondrial damage, followed by ferroptosis (Figure 1).

In summary, first, the failure of antioxidative defense under cystine starvation includes not only decreased GSH biosynthesis but also increased consumption of GSH and GPX4 to eliminate the large amounts of ROS resulting from glutaminolysis and iron overload. Second, the possible interaction between ferroptosis and mitochondria is that mitochondrial damage derived from the ROS burst induces ferroptosis under cysteine starvation. Third, it is of interest whether ferroptosis shares common pathways with mitochondrial damage or, to put it another way, whether ferroptosis forms an upstream or downstream relationship with mitochondrial damage, similar to the connection between mitochondrial damage and apoptosis. The ambivalent conclusions about the role of ETC-ROS in ferroptosis may be due to the use of testing methods that are not appropriate for cells with mitochondrial dysfunction, such as alamarBlue staining and the MTT assay, because these methods require mitochondrial enzymes to alter nicotinamide adenine dinucleotide (NADH) levels in order to judge cell death. The above uncertainties require further exploration and evidence.

### 4.2. ER Stress Initiation and UPR Protection

**Figure 2:**
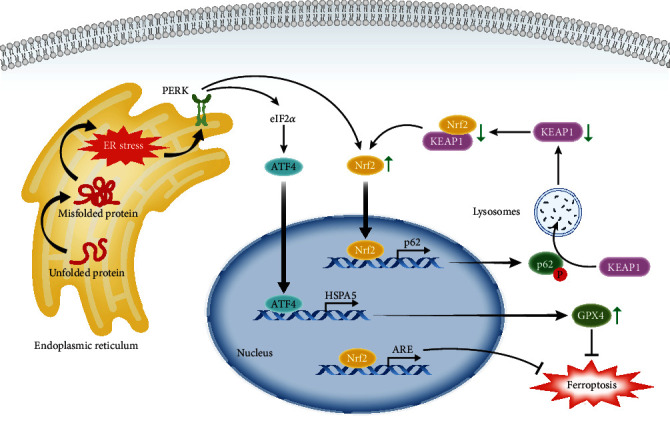
ER stress retards ferroptosis through the transcription factors ATF4 and Nrf2. Unfolded protein forms misfolded protein via oxidative protein folding (OPF) under excess ROS, initiating ER stress. Under ER stress, the activated sensor protein kinase RNA-like ER kinase (PERK) elicits the unfolded protein response (UPR), inducing transcriptional effects of nuclear factor E2-related factor 2 (Nrf2) and activating transcription factor 4 (ATF4) to resist ferroptosis. The resistant effects benefit from transcriptional production, including the antioxidant response element (ARE), the positive feedback loop related to sequestosome 1 (p62), and the increasing activity and expression of GPX4 related to heat shock protein family A member 5 (HSPA5). Relevant abbreviations: eIF2*α*: eukaryotic translation initiation factor 2*α*; KEAP1: Kelch-like ECH-associated protein 1.

Oxidative protein folding (OPF), the most common form of posttranslational protein modification in the endoplasmic reticulum (ER), induces the accumulation of misfolded proteins under excess ROS production, triggering ER stress [58]. Under ER stress, the unfolded protein response (UPR) is launched through three types of sensors, inositol-requiring protein 1*α* (IRE1*α*), protein kinase RNA-like ER kinase (PERK), and activating transcription factor 6*α* (ATF6*α*), to repair ER homeostasis, and the activated PERK is related to ferroptosis [59]. The system x_C_^−^ inhibitors erastin and sorafenib activated the PERK pathway, in which we could observe the phosphorylation of eukaryotic translation initiation factor 2*α* (eIF2*α*) and the increase in the activating transcription factor 4 (ATF4) in HT-1080 cells; however, the upregulation of CHAC1, a gene associated with ER stress, could be elicited only by system x_C_^−^ inhibitors [16] (Figure 2). That is, the inducers elicit ferroptosis by obstructing system x_C_^−^ and concomitantly initiating ER stress, and CHAC1 may be a ferroptotic pharmacodynamic marker. Moreover, while we know that excess ROS in the ER are derived from OPF and ETC-ROS [58], whether and how ROS accumulation, which is a core factor in ferroptosis, participates in ER stress and UPR remain to be explored.

Notably, actinomycin D, an inhibitor of transcription that can reverse the changes in these ATF4-related genes, delayed but did not reverse erastin-induced ferroptosis [16]. Furthermore, siRNA silencing of PERK and ATF4 increased lipid ROS and MDA in glioma cells, which could be prevented by DFO; conversely, upregulation of heat shock protein family A member 5 (HSPA5), which is the transcription product of ATF4, was synchronous with increasing expression and activity of GPX4 [59]. This attests to the protective and negative feedback mechanism of the PERK-ATF4-HSPA5-GPX4 pathway in dihydroartemisinin-induced ferroptosis. In addition, Nrf2 can be stimulated by activated PERK to form a positive feedback loop associated with sequestosome 1 (p62/SQSTM1) to promote ARE expression [60, 61]. In more detail, this feedback loop links through Kelch-like ECH-associated protein 1 (KEAP1) depletion mediated by phosphorylated p62 and the reduction of KEAP1/Nrf2 heterodimers, and thus, Nrf2 increases further [58] (Figure 2). Overall, the UPR response to ER stress can slow but not prevent the progress of ferroptosis via the excitation of HSPA5 and Nrf2; that is, UPR activity is a protective element that regulates ferroptosis. Interestingly, erastin or ART did not induce ferroptosis but apoptosis when cocultivated with TNF-related apoptosis-inducing ligand (TRAIL), and the results showed that the upregulation of ER stress markers, including ATF4, CHAC1, HSPA5, CHOP, and PUMA, by erastin treatment was not restored by ferroptosis inhibitors [62]. That is, the failure of erastin to elicit ferroptosis may be associated with ER stress and the protective effect of the UPR. The persistent excitation of the UPR promoted apoptosis by removing inhibition of B-cell lymphoma 2 (Bcl-2), which functions with Bcl-2-associated X protein/Bcl-2 homologous antagonist killer (Bax/Bak) via upregulation of the CHOP-PUMA axis located downstream of ATF4. Concomitantly, TRAIL-DRs (death receptors) promoted the transformation of the proapoptotic protein BID to truncated BID (tBID), permitting insertion of Bax/Bak into the mitochondria to promote mitochondrial permeabilization and ultimately leading to apoptosis [62]. That is, erastin-induced apoptosis consists of the activation of BID mediated by TRAIL-DRs and the disinhibition of Bax/Bak mediated by ER stress. We can deduce that ferroptotic inducers cause ER stress, which both protects against ferroptosis and induces apoptosis, resulting in the loss of the ability of these agents to induce ferroptosis. Antiapoptosis and antiferroptosis, both of which exist concurrently, may partially explain drug resistance in tumors. The remaining questions are as follows: whether resistance to system x_C_^−^ inhibitors in antiferroptotic tumors involves ER stress; whether ferroptotic sensitivity in tumors can be evaluated using ER stress markers; and whether combination therapy, including the regulation of ER stress, can be exploited to treat ferroptosis-associated diseases.

### 4.3. Adjustment of Autophagy to Ferroptosis

The process of autophagy has three steps: (i) phagophores derived from the membrane of the ER, Golgi bodies, and mitochondria selectively engulf contents in the cytoplasm; (ii) phagophores lengthen and seal into autophagosomes with double-membrane structures; and (iii) autophagosomes and lysosomes fuse into autolysosomes to enable degradation of the contents by hydrolase [63]. Many studies have demonstrated that autophagy is associated with ferroptosis.

#### 4.3.1. Ferritinophagy

**Figure 3:**
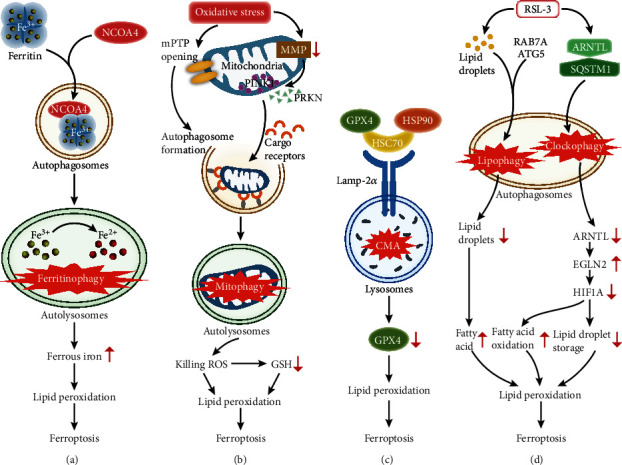
Autophagy exerts vital effects on ferroptosis via multiple mechanisms. (a) Ferritinophagy, the NCOA4-dependent degradation of ferritin, accumulates ferrous iron to induce ferroptosis. (b) Mitophagy, the degradation of impaired mitochondria via the gathering of PINK1 and PRKN as well as the transport of cargo receptors, produces “killing ROS” to advance ferroptosis. (c) CMA degrades its substrate GPX4 through the interaction between Lamp-2a located in lysosomes and the GPX4-HSC70-HSP90 trimer, resulting in ferroptosis. (d) Lipophagy, the RAB7A- and ATG5-dependent degradation of lipid droplets, increases the level of fatty acids. Clockophagy, the SQSTM1-dependent degradation of ARNTL, changes the status of fatty acid oxidation and lipid droplet storage by decreasing HIF1A after increasing EGLN2. Both lipophagy and clockophagy trigger abnormal lipid metabolism to regulate ferroptosis. Relevant abbreviations: NCOA4: nuclear receptor coactivator 4; PINK1: PTEN-induced kinase 1; PRKN: parkin RBR E3 ubiquitin protein ligase; CMA: chaperone-mediated autophagy; HSC70: heat shock cognate 70; HSP90: heat shock protein 90; Lamp-2a: lysosome-associated membrane protein type 2a; RAB7A: a member of the RAS oncogene family; ATG5: autophagy-related 5; ARNTL: aryl hydrocarbon receptor nuclear translocator-like protein 1; SQSTM1: sequestosome 1; EGLN2: egl nine homolog 2; HIF1A: hypoxia-inducible factor 1 subunit alpha.

Ferritin degradation mediated by nuclear receptor coactivator 4 (NCOA4) in autolysosomes produces large amounts of ferrous iron, named ferritinophagy (Figure 3). Different from previous research, Jiang Xuejun's lab showed that the excess iron derived from ferritinophagy contributed to ferroptosis. Based on these results, they proposed that autophagy greatly inhibits the early stage of ferroptotic process but not the advanced stage or potent inducement, which would explain why autophagy was not observed in early ferroptosis research [64]. The expression of ELAV-like RNA-binding protein (ELAVL1) increases when ferroptotic inducers (e.g., erastin and BSO) inhibit the ubiquitin-proteasome pathway, enhancing the stability of BECN1/Beclin1, which participates in LC3II and autophagosome formation [65]. However, ubiquitination of the RNA-binding protein ZFP36 increases the multimeric conjugated complex ATG12-ATG5-ATG16L1 in the phagophore [66]. Both increasing ELAVL1 and decreasing ZFP36 trigger ferritinophagy. In addition, BECN1 elicits autophagy-independent ferroptosis by forming the BECN1-SLC7A11 complex to inactivate system x_C_^−^ [67]. In contrast to the protective effects of p62 and HSPA5 against ferroptosis, the binding immunoglobulin protein (BiP), also known as HSPA5, shifts from the ER into the cytoplasm under ER stress to interact with p62, and then, phosphorylated p62 combines with LC3II to activate ferritinophagy [59, 68]. Moreover, mitochondrial ROS (MtROS) accumulation under mitochondrial impairment by arsenic also triggers NCOA4-mediated ferroptosis [69]. In brief, RNA-binding protein (e.g., ELAVL1 and ZFP36), ER stress (e.g., p62), and mitochondrial oxidative stress (e.g., MtROS) have pivotal effects on ferritinophagy. The underlying regulatory mechanisms and application prospects of the contradictory effects of p62 and HSPA5 remain to be probed.

#### 4.3.2. Mitophagy

Mitophagy is the process by which cargo receptors assemble in the outer mitochondrial membrane after PTEN-induced kinase 1 (PINK1) accumulation and parkin RBR E3 ubiquitin protein ligase (PRKN) collection at mitochondria to degrade impaired mitochondria [70]. Mitophagy is induced by mPTP opening-mediated autophagosome formation and MMP depolarization under the complex I inhibitor BAY, and then, “killing ROS” accumulate to depress GSH and trigger lipid peroxidation and ferroptosis [71]. In summary, under “killing ROS” generated by mitophagy, the increases in GSH consumption and lipid peroxidation are regarded as central events to link ferroptosis (Figure 3).

#### 4.3.3. Chaperone-Mediated Autophagy (CMA)

Heat shock cognate 70 (HSC70) and heat shock protein 90 (HSP90), as molecular chaperones, bind the CMA substrate GPX4 to form the GPX4-HSC70-HSP90 trimer, which interacts with lysosome-associated membrane protein type 2a (Lamp-2a) located at the lysosomal membrane. HSC70 combines with Lamp-2a, and HSP90 stabilizes Lamp-2a [72]. Subsequent degradation of GPX4 triggers ferroptosis [72]. In summary, GPX4 degradation is the crossroads between CMA and ferroptosis (Figure 3).

#### 4.3.4. Lipophagy and Clockophagy

Lipophagy and clockophagy use abnormal lipid storage and degradation to execute RSL-3-induced ferroptosis. In lipophagy, increasing fatty acid due to RAB7A- and ATG5-dependent lipid degradation induces ferroptosis [73]. In clockophagy, the core circadian clock protein ARNTL degrades after bonding SQSTM1, causing hypoxia-inducible factor 1 subunit alpha (HIF1A) decomposition due to egl nine homolog 2 (EGLN2) upregulation and consequently ferroptosis [74, 75]. HIF1A positively regulates the storage of lipid droplets and negatively regulates fatty acid oxidation [74]. In summary, RSL-3 causes lipophagy and clockophagy to trigger abnormal lipid metabolism, ensuing lipid peroxidation and ferroptosis (Figure 3).

In summary, autophagy, regarded as the upstream regulator of ferroptosis, has various regulatory effects, including ferritinophagy, mitophagy, CMA, lipophagy, and clockophagy. Advances in research on the mechanism of autophagy-dependent ferroptosis provide innovative tactics and underpin the extant strategies for ferroptotic therapies.

### 4.4. DAMP Release after Ferroptosis

The mechanisms of ferroptosis have been discussed above; hereafter, the mechanisms of ferroptosis-induced tissue injury and tumorigenesis will be addressed as follows. Damage-associated molecular patterns (DAMPs) have garnered widespread attention as significant participants in these mechanisms.

DAMPs are released after ferroptosis and interact with toll-like receptor (TLR-4), which is expressed in endothelial cells; subsequently, the collection and adhesion of neutrophils initiate inflammation by increasing the expression of interferon (IFN-*α*) via TIR-domain-containing adapter-inducing interferon-*β* (Trif) [76]. In addition, the accumulation of ROS in ferroptosis retards the function of dimethylarginine dimethylaminohydrolase (DDAHII) in degrading asymmetric dimethylarginine (ADMA), and the generation of the anti-inflammatory factor nitric oxide (NO) is decreased because excess ADMA competitively binds with endogenous nitric oxide synthase (eNOS) [54]. In short, ferroptosis initiates inflammation via the TLR4/Trif/IFN-*α* signaling pathway, and the accumulation of ROS activates the ROS/ADMA/DDAHII/eNOS/NO pathway to impair the anti-inflammatory effect of NO. The corollary is that ferroptosis is one of the critical causes of tissue inflammatory injury.

Under oxidative stress, after autophagy-dependent ferroptosis, melanoma cells release DAMP KRAS^G12D^ protein, which is transported to macrophages via exosomes. KRAS^G12D^ protein is recognized through advanced glycosylation end product-specific receptor (AGER) and is taken up into macrophages [77]. After that, the transcription of related genes for fatty acid *β*-oxidation is activated by phosphorylated STAT3, and *β*-oxidation leads to procarcinogenic macrophage M2 polarization, promoting tumorigenesis and tumor progression [77]. In short, KRAS^G12D^-mediated macrophage M2 polarization via the AGER-STAT3 pathway has oncogenic effects, stimulating tumor growth.

It is reasonable to conjecture that the release of DAMPs after ferroptosis is closely related to the occurrence and development of pathological processes.

## 5. The Links between Ferroptosis and Disorders

### 5.1. Tumors

As an essential cause of increasing morbidity and mortality with each passing year, the intrinsic mechanism of tumors is of immense research interest. Limiting the activity of system x_C_^−^ by ferroptotic inducers can improve diffuse large B-cell lymphoma (DLBCL) [78]. Renal cell carcinoma (RCC) is more sensitive to ferroptosis under an iron overload state and low GPX4 levels [12]. Preventing the compensatory increase in SLC7A11 in ferroptosis may be a target for combination therapy for tumors. IFN-*γ* released by activated CD8^+^ T cells in cancer immunotherapy inhibits the expression of SLC7A11 to enhance sensitivity to ferroptosis inducers, thereby limiting tumor proliferation [79]. p53^3KR^, an acetylation-defective mutant, represses the transcription of SLC7A11 to induce ferroptosis under higher ROS levels in tumor tissue, subsequently inhibiting tumor growth [80]. In addition, single-cell transcriptome analysis has shown that ferroptosis of granulocytes may be responsible for the high proportion of granulocytes in colorectal cancer liver metastasis samples, showing that the tumor microenvironment is another crucial target in oncotherapy that is closely connected to ferroptosis [81]. Clinical anticarcinogens containing ferroptosis inducers have widespread applications in iron-rich tumor tissues, including hepatoma and pancreatic carcinoma [16, 31, 54]. Combination therapy with piperlongumine, cotylenin A, and SAS has shown effectiveness as a treatment for pancreatic carcinoma via induction of ferroptosis [82]. Pancreatic ductal adenocarcinoma (PDAC) has higher cysteine metabolism requirements than normal tissues for synthesizing GSH and converting coenzyme A (CoA) to cope with the by-product ROS. Cyst(e)inase, which depletes cystine/cysteine, rescues viability in mice with PDAC by promoting ferroptosis [83]. Lipid droplets and mitochondrial damage can also be observed in vivo [83]. Similar to high-level cysteine metabolism, HSPA5 also promotes PDAC progression by resisting ferroptosis [84]. This shows that curtailing cysteine and HSPA5 to treat pancreatic tumors is feasible and promising, and mitochondrial damage, ER stress, and disorganized lipid and amino acid metabolism have been unexpectedly observed in PDAC. Siramesine and lapatinib alter iron regulation to treat breast cancer [85], while SAS impedes the growth of triple-negative breast cancer through inhibition of SLC7A11 [86, 87]. In all cases considered, ferroptosis suggests possible efficient precautionary means and therapies to be exploited for curtailing tumor morbidity and mortality. The clinical transformation of ferroptosis targets is still in the continuously exploratory stage.

### 5.2. Cardiovascular System Disease

Mice lacking ferroportin in cardiomyocytes showed aggravated dilated cardiomyopathy when the level of cardiac iron was increased. This change was improved by a low-iron diet [88]. Fang et al. verified that ferritin deficiency in cardiomyocytes promoted mild cardiac damage in mice, which progressed to hypertrophic cardiomyopathy when the mice were fed a high-iron diet. These cardiac injuries were reversed by SLC7A11 overexpression [89]. DOX mediates iron-replete ferroptosis via the nuclear translocation of Nrf2 and decomposition of HO-1 to induce cardiomyopathy and heart failure (HF) [29]. There are reasons to believe that the development of cardiomyopathy and severe cardiac dysfunction can be ascribed to iron overload in cardiomyocytes. In addition, quantitative proteomic analyses have shown that GPX4 levels decline in the early and middle phases of MI, promoting cardiomyocyte ferroptosis; subsequently, fiber tissues with no systolic properties are substituted for inactivated cardiomyocytes [90]. In this case, the patient will even progress into HF [90, 91]. Among clinical therapies for HF patients, iron chelation has been recommended for HF patients without iron deficiency in recent studies [92]. Although a series of clinical tests have demonstrated that intravenous iron supplementation is beneficial for HF patients with iron deficiency, this therapy does not seem well designed to limit latent side effects, including local iron overload and myocardial fibrosis [92]. In general, the role of iron overload as an essential feature in ferroptosis and consequently in cardiovascular damage should not be overlooked. In further explorations of therapeutic targets, the restoration of iron homeostasis in the heart and interruption of ferroptosis should be taken into account.

### 5.3. Ischemia/Reperfusion Injury (IRI)

Cardiomyocyte death in the left ventricle was observed after reperfusion in mice undergoing heart transplantation, and increases in creatine kinase release and PUFA peroxide product formation were observed using Langendorff preparations to mimic IRI. Infarct area enlargement and worsening cardiac function in myocardial infarction (MI) were observed during IRI. Notably, all of the above damage was mitigated by Fer-1 treatment [76]. Mechanistically, IRI can elicit the release of DAMPs to initiate inflammation and then induce tissue damage, which is consistent with the above findings. The effects of IRI-related acute renal failure (AKF) mediated by GPX4 knockdown and hepatic injury caused by IRI could be repaired by ferroptosis inhibitor treatment [11]. Ferroptosis has also been confirmed to induce intestinal ischemia/reperfusion-induced acute lung injury [93]. Accordingly, ferroptosis contributes to regulating the pathological process after IRI and supplies therapeutic targets for IRI-related visceral injury.

Instances such as these are as common. Sorafenib promotes ferritinophagy-mediated ferroptosis to retard hepatic fibrosis and cirrhosis [65]. The DAMPs released by ferroptosis in epithelial cells induce necroinflammation, resulting in a series of clinical phenotypes in chronic obstructive pulmonary disease (COPD) [94]. Improvements in intracerebral hemorrhage [36], Huntington's disease, and periventricular leukomalacia [95] can be achieved by subverting ferroptosis. We speculate that targeting ferroptosis holds potential for multiple pathophysiological processes, which awaits further exploration and potential clinical application.

## 6. Conclusions and Prospects

In light of the links between ferroptosis and multiple diseases in recent reports, this overview summarizes the discovery, characterization, and regulatory mechanisms of ferroptosis together with its crosstalk with other responses. Herein, we shed light on the mechanisms of some ferroptosis-related compounds and emphasize the crosstalk between ferroptosis and other responses, including mitochondrial damage, ER stress, autophagy, and the release of DAMPs, to reveal the intersections of the regulatory mechanisms of these processes. We note the striking paradoxical effects of controversial elements, including Nrf2, p62, and HSPA5, and summarize the known pathways related to the interaction between the TCA cycle, oxidative phosphorylation or fatty acid metabolism, and ferroptosis, in which *α*-KG is a central molecule.

We briefly address plausible speculations regarding the potential clinical application of ferroptosis. In terms of oncotherapy, therapeutic failure may be partly caused by the protective effect of ER stress; correspondingly, markers of ER stress may be used to evaluate efficacy. In contrast, ferroptosis exhibits tumorigenicity due to the release of DAMPs. In addition, FSP1, GCH1, and IFN-*γ* are innovative targets that can be exploited for tumor resistance. In terms of inflammation therapy, given that ferroptosis initiates inflammation via DAMP release, inflammatory diseases may be improved by impeding ferroptosis. In terms of metabolic disorders, we should consider whether ferroptosis can introduce a new tactical direction in view of its intersections with metabolism, including energy, fat, and amino acid metabolism. In conclusion, multidirectional insight into the mechanism and crosstalk of ferroptosis has great significance for disorder prevention and treatment, and the controversial elements awaiting exploration provide clues in the search for methods of regulating ferroptosis.
